# A Validated LC-MS/MS Method for Simultaneous Determination of Cortisol and Cortisone in Grey Wolf Hair for Application in Ecological Studies

**DOI:** 10.3390/molecules31142420

**Published:** 2026-07-10

**Authors:** Arkadiusz Jastrzębski, Kinga Ożga-Wybranowska, Rafał Łopucki, Sabina Nowak, Robert W. Mysłajek, Ilona Sadok

**Affiliations:** 1 Department of Biomedicine and Environmental Research, Institute of Biological Sciences, Faculty of Medicine, The John Paul II Catholic University of Lublin, Konstantynów 1J, 20-708 Lublin, Poland; arkadiusz.jastrzebski@kul.pl (A.J.); kinga.ozga@kul.pl (K.O.-W.); lopucki@kul.pl (R.Ł.); 2 Department of Animal Ecology and Evolution, Institute of Ecology, Faculty of Biology, University of Warsaw, Żwirki i Wigury 101, 02-089 Warszawa, Poland; s.pieruzek-nowak@uw.edu.pl (S.N.); r.myslajek@uw.edu.pl (R.W.M.); 3 Association for Nature “Wolf”, Cynkowa 4, 34-324 Twardorzeczka, Poland; 4 Department of Biomedical and Analytical Chemistry, Institute of Biological Sciences, Faculty of Medicine, The John Paul II Catholic University of Lublin, Konstantynów 1J, 20-708 Lublin, Poland

**Keywords:** glucocorticoids, ELISA, method validation, steroid hormones, conservation biology, wildlife, stress, *Canis lupus*

## Abstract

Hair is an easily obtainable, non-invasive biomatrix that allows for the assessment of long-term physiological responses to environmental and anthropogenic stressors in wildlife populations. Herein, an ultra-high-performance liquid chromatography–electrospray ionization–tandem mass spectrometry (UHPLC-ESI-MS/MS) method was validated to verify its suitability for the simultaneous determination of cortisol (CORT) and its metabolite, cortisone (CORN), in hair samples collected from wild-living grey wolves (*Canis lupus*). Hair samples were extracted with methanol and purified using solid-phase extraction on Strata-X cartridges, which enabled effective mitigation of matrix effects. Data for the glucocorticoids were normalized using internal standards. The method demonstrated good linearity for the target stress hormones, with satisfactory precision (RSD < 15%) and limits of quantification of 4.13 pg/mg for CORT and 2.49 pg/mg for CORN in the hair matrix. Analysis of authentic wolf hair samples revealed CORT and CORN concentrations in the ranges of <4.13–11.86 pg/mg and <2.49–3.67 pg/mg, respectively. The CORT results showed a strong positive correlation with those obtained using enzymatic immunoassays. The method may be applied to assess the impact of stressors on the welfare of wolves, e.g., providing a useful tool for monitoring recovering European populations as they face new challenges associated with expansion into potentially suboptimal habitats.

## 1. Introduction

Cortisol (CORT) and cortisone (CORN) are closely related glucocorticoids widely used as biomarkers of stress in humans and animals [1,2,3,4,5]. They play important roles in metabolism, stress response, and immune regulation [1]. CORT is the biologically active glucocorticoid synthesized in the adrenal cortex and released in response to stress via activation of the hypothalamic–pituitary–adrenal (HPA) axis [2,3]. It is distributed throughout the body via the bloodstream and exerts metabolic, anti-inflammatory, and immunosuppressive effects [4,5]. CORT also follows a circadian rhythm, with peak levels typically observed in the early morning [6]. In contrast, CORN is generally considered an inactive or much less active metabolite of CORT. It is formed from CORT by 11β-hydroxysteroid dehydrogenase type-2 (11β-HSD2) and can be converted back to active CORT by 11β-HSD type-1 [7,8]. This reversible interconversion regulates local glucocorticoid activity and is therefore relevant for the interpretation of analytical results. Measurement of CORT and CORN in biological matrices such as blood, saliva, feces, hair, or urine is widely used in medical and veterinary research [1,9,10,11,12]. Simultaneous determination of both analytes may improve the interpretation of glucocorticoid profiles in complex biological matrices such as hair.

Hair, particularly its proximal segment, is often used as a retrospective matrix reflecting glucocorticoid accumulation over an extended period, although the exact time window may vary among species [13]. Hair cortisol is assumed to reflect the free, biologically active fraction of CORT more accurately than total cortisol measured in plasma or serum [14]. In addition, because hair collection is non-invasive, it allows long-term stress exposure to be assessed without inducing additional stress or altering the physiological state of the study subject [14]. However, the available data on long-term glucocorticoid stability in hair remain inconsistent. While recent controlled studies have demonstrated significant declines in CORT and CORN concentrations after as little as six months of storage at room temperature [15], other investigations suggest that hair may remain a relatively stable matrix for endocrine assessment over much longer periods, potentially up to five years [16]. Furthermore, exposure to natural sunlight and UV radiation has been shown to substantially reduce glucocorticoid concentrations in hair, highlighting the importance of appropriate sample storage conditions [17].

Immunoassays based on antibodies are widely used for monitoring stress hormones [18,19,20,21,22]. ELISA-based assays are characterized by high sensitivity and reliability, enabling detection of concentrations ranging from sub-pg/mL to ng/mL; however, this method has certain limitations [4]. These include limited specificity due to cross-reactivity with other steroid hormones of similar chemical structure [23]. This limitation is particularly relevant in hair glucocorticoid analysis, where assay choice and antibody specificity may substantially affect measured concentrations and even lead to erroneous biological interpretation [24]. Furthermore, commercially available ELISA kits may differ in antibody specificity and inter-assay variability, potentially contributing to discrepancies in CORT concentrations measured in hair across studies [25]. For the determination of glucocorticoid levels in biological samples, chromatographic techniques, particularly those coupled with mass spectrometry, are gaining increasing importance. Among them, liquid chromatography coupled with tandem mass spectrometry (LC-MS/MS) has found broad application in monitoring a variety of corticosteroids for assessment of stress levels in humans and animals [22,26,27,28,29,30,31,32,33]. A major advantage of this hybrid technique is its versatility and its ability to simultaneously detect and quantify multiple target analytes within a single analytical run under the same measurement conditions. Through appropriate selection of sample preparation conditions, LC-MS/MS methods enable the determination of CORT and CORN in a wide range of biological matrices, including blood as well as samples such as hair [22,26,28,30,33], faeces [32], urine [34], and saliva [27,35], which can be collected in a less invasive manner. This is particularly important in studies assessing the effects of environmental stressors on animal welfare, especially in rare, endangered, or free-living species, for which blood sampling may be difficult, impractical, or potentially stressful, making less invasive matrices a valuable alternative.

Despite the undeniable advantages of LC-MS/MS approaches, their key limitation is the need to carefully optimize sample preparation conditions to minimize analyte losses while effectively removing matrix components that may affect quantitative results [32,36,37]. In particular, attention should be paid to the so-called matrix effect (ME) [38]. This phenomenon is especially relevant in analyses performed using systems equipped with an electrospray ionization source [38]. ME results from changes in the ionization efficiency of analytes caused by the presence of co-eluting matrix components, often leading to signal suppression or enhancement and, consequently, to biassed analytical results [39].

ME can be compensated for using strategies such as appropriate selection of chromatographic separation conditions, optimization of ion source parameters, sample dilution, incorporation of a clean-up step into the sample preparation protocol, or the use of internal standard-based normalization [37,38]. In particular, ME may vary within the same type of biological matrix, e.g., hair, depending on the animal species from which the sample originates [28]. In practice, this means that the use of a validated LC-MS/MS method dedicated to the analysis of CORT and CORN in hair from a given animal species does not necessarily ensure the same analytical performance (e.g., limits of detection (LOD), lower limit of quantification (LLOQ), recoveries (R_E_), accuracy, and precision) when applied to hair from another species [28]. Therefore, species-specific validation, or at least verification and appropriate adaptation of existing protocols, is particularly important to ensure the reliability of the obtained results.

The grey wolf (*Canis lupus*), a large carnivore of high ecological and conservation relevance, is currently undergoing population recovery and range expansion in Europe [40,41]. As wolves recolonize landscapes increasingly shaped by human activity, they may face new ecological and anthropogenic challenges, including habitat fragmentation, human disturbance, illegal killing, and conflicts related to anthropogenic food sources [42,43,44]. These processes create a growing need for reliable, non-invasive tools to monitor physiological stress and welfare in free-living wolf populations [45]. Previous studies investigating hair cortisol in grey wolves have relied exclusively on ELISA [45,46]. Similarly, CORT determination in hair has been investigated in closely related canid species, including domestic dogs (*Canis familiaris*) and coyotes (*Canis latrans*), where immunoassays have also been predominantly employed [47,48,49,50]. To the best of our knowledge, no validated ultra-high-performance liquid chromatography–electrospray ionization–tandem mass spectrometry (UHPLC-ESI-MS/MS) method has yet been reported for the simultaneous determination of CORT and CORN in wolf hair. To address this gap, the present study provides the first species-specific validation of aUHPLC-ESI-MS/MS method for the simultaneous determination of CORT and CORN in grey wolf hair. Although our previously developed analytical workflow had been successfully applied to hair samples from the European bison (*Bison bonasus*), European hamster (*Cricetus cricetus*), and Eurasian red squirrel (*Sciurus vulgaris*) [26,28], its applicability to wolf hair could not be assumed because hair matrix composition and associated matrix effects may vary considerably among species. Therefore, the method was comprehensively re-optimized and validated specifically for grey wolf hair, with particular emphasis on evaluating ME and ensuring reliable quantification using isotopically labelled internal standards (ILIS). The applicability of the method was demonstrated by analyzing authentic hair samples from free-living wolves. Additionally, CORT concentrations determined by LC-MS/MS were directly compared with those obtained using ELISA to assess the consistency between the two analytical approaches.

## 2. Results and Discussion

**Figure 1:**
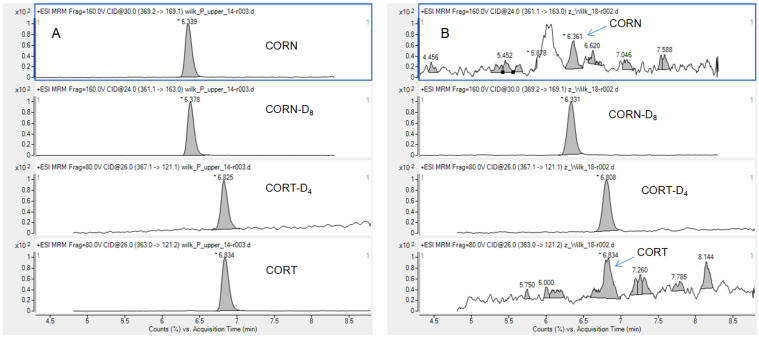
Representative dynamic multiple reaction monitoring (dMRM) chromatograms showing the retention times of cortisol (CORT), cortisone (CORN), and their deuterated internal standards in (**A**) a quality control sample containing high levels of glucocorticoids and (**B**) hair extract from the grey wolf. The dot above the retention time is a software-generated peak marker.

The UHPLC-ESI-MS/MS protocol previously developed for the determination of CORN, CORT, and corticosterone in hair from wild animals [28] was modified to adapt it to the wolf hair matrix. The general analytical workflow included hair prewashing with isopropanol to remove external contaminants typically present in samples collected from wild animals, homogenization, extraction in the presence of ILIS, sample clean-up using solid-phase extraction (SPE), and sample concentration before UHPLC-ESI-MS/MS analysis. The most important method modifications introduced in the present study included changing the analytical column and implementing result normalization using two ILIS corresponding to the target glucocorticoids. Ions were monitored in dynamic multiple reaction monitoring (dMRM) mode, and representative data acquired from quality control (QC) samples are presented in Figure 1. The addition of ILIS or other internal standards at the earliest stages of sample preparation represents an effective strategy for ensuring accurate and reliable determination of target analytes in complex biological matrices and for mitigating ME [28,31,33]. The improved method was further validated to assess its reliability and robustness, and its applicability was evaluated in two stages: (1) through the analysis of hair samples from wild-living wolves and (2) by assessing its consistency with ELISA assay results.

### 2.1. Method Validation

#### 2.1.1. Method Linearity, LOD, and LLOQ

The LOD and LLOQ values, together with the linear ranges of the calibration curves for each target glucocorticoid, were determined in the presence of the grey wolf hair matrix (Table 1). The developed method enabled the quantification of CORT and CORN at levels as low as 4.13 and 2.49 pg/mg, respectively. Matrix-matched calibration curves demonstrated good linearity over broad concentration ranges, covering low pg/mg to low ng/mg levels for both analytes.

In a previous study involving hair from the European bison and red squirrel, analyzed using a similar sample preparation protocol, the determined LLOQ values for CORT ranged from 5.12 to 90.12 pg/mg, while those for CORN ranged from 3.87 to 34.76 pg/mg, depending on the species [28]. Therefore, in the present study, slightly lower LLOQs for both target steroid hormones were achieved in the wolf hair matrix (Table 1). The analytical sensitivity of the proposed method could potentially be further improved by reducing the final reconstitution volume of the extract. However, concentrating the extract would also increase the concentration of co-extracted matrix constituents, which may enhance ME and affect the quantitative performance of the method. Therefore, implementation of this approach would require comprehensive re-validation, including re-evaluation of matrix effects, precision, accuracy, and the LLOQs for both analytes.

By comparison, another LC-MS/MS method employing different sample treatment and analytical conditions achieved comparable LOD for CORT in horse hair (1 pg/mg), with 25 mg of hair required for the analysis [30]. In a study focused on polar bear (*Ursus maritimus*) hair, lower LODs for CORT (0.13 pg/mg) and CORN (0.05 pg/mg) were reported for an LC-MS/MS method compared with the values obtained in the present study [51]. However, it was not clearly stated whether these values were determined in the hair matrix, as in the present study, or calculated using standard solutions prepared in pure solvent.

In human hair studies, LC-MS/MS approaches typically achieve lower LLOQs for CORT and CORN in the range of 0.25–0.5 pg/mg, which may be attributed to differences in matrix composition as well as in the applied analytical conditions [31,33].

Several multi-analyte LC-MS/MS approaches that allow the simultaneous determination of CORT and CORN along with other biologically active compounds have already been developed [31,33,52]. For example, one approach enabled the determination of CORT and CORN in human hair at very low detection limits (0.25 pg/mg), simultaneously with multiple estrogens, androgens, and progestogens [33]. However, this protocol requires chemical derivatization of keto groups using Girard P reagent and is associated with a narrower linear dynamic range of the calibration curves [33]. Similarly, another LC-MS/MS-based protocol has been developed for the simultaneous determination of multiple steroid hormones and endocannabinoids in hair using methanolic extraction followed by automated clean-up procedures such as supported liquid extraction [31]. In another study, CORT and CORN were determined by LC-MS/MS simultaneously with multiple illicit drugs without a purification step for human hair extracts [52]. Although this method provided lower LOQs for CORT and CORN (1.6 and 1.2 pg/mg, respectively), the calibration curves exhibited narrower linear ranges (1.25–250 pg/mg) than those obtained in the present study [52].

Available protocols for CORT and CORN determination in hair frequently require 25–50 mg of sample for extraction [22,30,31,33], while the present method uses 35 mg of hair, which falls within this range and is suitable for applications where sample availability is limited. Additionally, in contrast to previously reported protocols, the method proposed in this study was specifically optimized for the reliable quantification of CORT and CORN in a wildlife-relevant matrix (wolf hair), offering a simplified workflow without derivatization steps and employing SPE with commercially available cartridges for extract clean-up. Compared with the LC-MS/MS method reported for the closely related domestic dog, the present protocol similarly employs SPE for sample clean-up but requires substantially less hair material (35 mg vs. 100 mg). In addition, the canine study did not include full analytical validation or determination of LLOQ values [49]. To the best of our knowledge, this is the first validated UHPLC-ESI-MS/MS method for the determination of CORT and CORN in grey wolf hair.

#### 2.1.2. Method Precision and Accuracy

Intraday precision was ≤8.2% for CORT and ≤9.6% for CORN, regardless of the concentration level evaluated (Table 2). Intraday precision was ≤13.0% for CORT and ≤11.4% for CORN. All precision values fell within the acceptable range, indicating that the method provides precise results for the determination of CORT and CORN in the grey wolf hair extracts. Furthermore, both intraday and interday accuracy ranged from 101.0% to 108.4% for CORT and from 100.9% to 109.3% for CORN (Table 2), confirming acceptable method accuracy across both low and high concentrations of the target glucocorticoids.

The satisfactory precision and accuracy observed in the present study may be attributed, at least in part, to the addition of ILIS at the early stages of sample preparation, which enables compensation for random and systematic errors occurring during sample handling and enhances the reliability of quantitative analysis [53,54]. Furthermore, ILIS co-eluting with the target analytes provides an additional reference supporting reliable peak identification during the analysis of complex biological matrices. Because ILIS exhibit nearly identical physicochemical properties and chromatographic behaviour to their corresponding unlabelled analytes, they are considered particularly valuable for improving the robustness and reproducibility of quantitative LC-MS/MS methods [54]. However, deuterium-labelled internal standards may exhibit unexpected behaviour, such as slightly different retention times, known as the isotope effect, or different recoveries compared with the corresponding analytes [54,55]. Method validation can help identify such issues. Another drawback of using ILIS is their high cost and, in some cases, limited availability [54].

#### 2.1.3. Recovery and Matrix Effect

For CORT, the R_E_ values ranged from 77.0 to 81.9% across all evaluated QC concentration ranges (Table 2). CORN exhibited higher R_E_ values, ranging from 80.0 to 96.4%. All obtained R_E_ values fell within the acceptable validation range. Previously, similar R_E_ values were observed for both analytes in the European hamster hair matrix [28] using the same sample preparation protocol for corticosterone. However, the R_E_ values for CORT reported here in grey wolf hair were lower than those previously reported for the European bison and red squirrel hair matrices [28]. For comparison, another UHPLC-ESI-MS/MS study conducted on human hair reported R_E_ values ranging from 77.1 to 84.3% for CORT and from 92.9 to 105% for CORN, using a protocol that required analyte derivatization [33]. Similar R_E_ values have also been reported for other LC-MS/MS methods employing different sample preparation protocols, with R_E_ values for CORT and CORN in human hair estimated at 86.9–89.9% and 84.7–87.7%, respectively [30].

Since ME is highly analyte-dependent [38], it was evaluated separately for each glucocorticoid at three concentration levels: LQC, MQC, and HQC. The applied hair extract clean-up strategy effectively reduced the influence of matrix components on the analytical responses of CORT and CORN (Table 2). The determined ME values ranged from 92.3 to 100.9%, irrespective of the target analyte or its concentration level in the QC sample. For comparison, using the same sample extraction and extract purification protocol, significant signal suppression for both CORT and CORN was previously observed in European bison and red squirrel hair matrices [28].

These observations confirm that the extent of ME may strongly depend on the biological origin of the hair matrix, even when identical sample preparation and instrumental conditions are applied. Differences in analytical responses during the analysis of hair extracts from different animal species, as well as between individuals of the same species, may result from variations in endogenous hair constituents such as lipids, pigments, proteins, and carbohydrates, but may also arise from the influence of external contamination, including environmental salts, surfactants, and other contaminants [38,56]. These endogenous and exogenous hair components may be co-extracted with CORT and CORN, and may substantially influence ionization efficiency during LC-MS/MS analysis, especially when ESI is used, as this ionization technique is particularly prone to ME [38]. Studies on human hair further highlight the critical role of sample preparation protocol selection, as reported ME values for CORT and CORN vary markedly between methods, ranging from negligible ME to substantial signal suppression in LC-MS/MS determinations of these glucocorticoids [30,33]. The satisfactory mitigation of ME observed in the present study may therefore result from both the introduction of the hair prewash step, which reduces external contamination associated with the hair fibre surface, and the effectiveness of the SPE clean-up procedure. The obtained results further emphasize the importance of species-specific validation of LC-MS/MS methods intended for glucocorticoid determination in hair samples from wild animals, in order to increase confidence in quantitative data for evaluating the impact of environmental stressors on animal welfare.

#### 2.1.4. Stability

In this study, the stability of CORT and CORN was evaluated by determining autosampler stability (S_A_) and freeze–thaw stability (S_FT_). The tests allowed the identification of potential bias in analytical results arising from analyte degradation caused by matrix components, short-term storage in the autosampler, or repeated freeze–thaw cycles. The S_A_ values ranged from 93.7% to 96.0% for CORT and from 93.9% to 98.4% for CORN. Freeze–thaw stability ranged from 91.1% to 97.4% for CORT and from 90.6% to 104.8% for CORN. Both stability parameters showed satisfactory values, as the results did not differ by more than ±10% from those obtained for freshly prepared samples, regardless of the analyte and QC concentration level. These results demonstrate adequate stability of CORT and CORN under both autosampler and freeze–thaw conditions, further confirming the suitability of the method for the quantitative analysis of hair samples. Nevertheless, the use of ILIS may further help compensate for variations in analytical response associated with matrix-related effects, analyte losses, or minor instability during sample handling and storage [53].

### 2.2. Authentic Samples Analysis

To confirm the applicability of the method, hair samples from nine free-living wolves were analyzed by UHPLC-ESI-MS/MS. The obtained results are summarized in Table 3, and representative chromatograms are presented in Figure 1B.

Among the analyzed samples, CORT was quantified in six samples at concentrations ranging from 4.22 to 11.86 pg/mg, whereas in three samples this glucocorticoid was detected but not quantified, as its concentration was below the LLOQ of 4.13 pg/mg. CORN signals were observed in all authentic samples; however, concentrations above the LLOQ were obtained only in four samples, ranging from 2.52 to 3.67 pg/mg.

It has been demonstrated that CORT concentrations measured in animal hair are affected by numerous biological and environmental factors, including the health status, body mass, sex, age, and habitat [57]. In addition, discrepancies among literature data may arise from differences in the applied analytical methodologies, especially hair homogenization procedures and the detection technique employed, such as LC-MS/MS or ELISA [24,51]. Nevertheless, the CORT concentrations determined in the present grey wolf samples are in agreement with previously published data [45,46]. For example, CORT concentrations ranging from 1.6 to 108.8 pg/mg (*n* = 259) were previously reported for lumbar guard hair collected from wolves representing four European populations: Iberian, Alpine, Dinaric–Balkan, and Scandinavian [45]. Another study conducted on wolves (*Canis lupus ligoni*) from Prince of Wales Island, AK, USA, showed CORT levels in guard hair ranging from 1.6 to 20.6 pg/mg (*n* = 23), and in undercoat ranging from 3.5 to 23.8 pg/mg (*n* = 25) [46]. These studies employed ELISA assay for CORT quantification, whereas CORN levels were not determined. The UHPLC-ESI-MS/MS method presented herein may therefore serve as a robust analytical approach capable of providing data not only on CORT but also on CORN, enabling a more comprehensive interpretation of the physiological status of animals.

### 2.3. Comparison of CORT Levels Determined by UHPLC-ESI-MS/MS and ELISA

**Figure 2:**
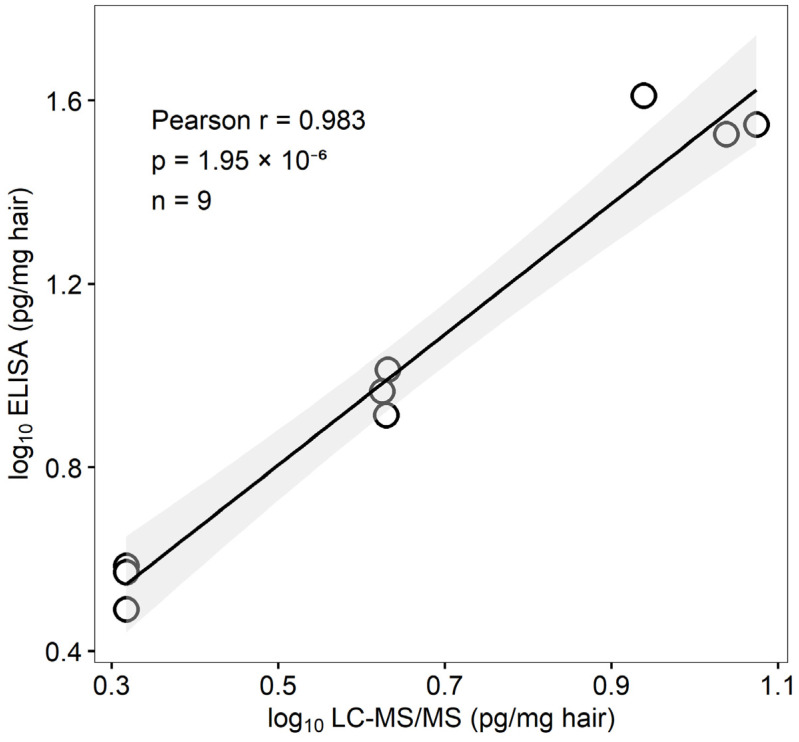
Correlation between log10-transformed hair cortisol concentrations in wild grey wolves determined by ELISA and UHPLC-ESI-MS/MS. Each point represents one individual sample.

The relationship between CORT concentrations determined using the UHPLC-ESI-MS/MS method presented herein and those obtained with the ELISA assay was evaluated using scatter plot analysis after log10 transformation. As shown in Figure 2, the results obtained by both techniques followed a similar trend. A strong positive correlation was observed between the two approaches, both in terms of Pearson correlation (r = 0.983, *p*< 0.001) and Spearman rank correlation (ρ = 0.915, *p* < 0.001), indicating that samples with higher CORT concentrations measured by UHPLC-ESI-MS/MS also tended to yield higher values when analyzed by ELISA. These findings support the applicability of the presented UHPLC-ESI-MS/MS method for the determination of CORT in grey wolf hair samples.

A discrepancy, reflected by higher CORT concentrations obtained using the ELISA assay, was observed particularly for samples containing CORT at high concentration levels. Similar overestimation of CORT concentrations by ELISA compared with LC-MS/MS has previously been reported [30,51]. For example, ELISA yielded approximately 1.6-fold higher CORT concentrations in horse hair samples than liquid chromatography coupled with hybrid high-resolution mass spectrometry (LC-HRMS/MS) [30]. This overestimation may result from the cross-reactivity of ELISA antibodies with structurally related endogenous corticosteroids present in the analyzed matrix [30,51]. In contrast, LC-MS/MS-based approaches offer superior analytical selectivity and enable more reliable quantification of CORT in hair samples [30,51]. Furthermore, LC-MS/MS-based methods enable the simultaneous determination of CORT and CORN, thereby providing a more comprehensive characterization of the steroid profile in hair samples than ELISA assays. Furthermore, studies in humans have shown that 11β-HSD2 is expressed in eccrine sweat glands, suggesting that the conversion of CORT to CORN may occur locally within the skin [58,59]. Consequently, the CORN concentration measured in hair may not solely reflect circulating cortisone levels but may also result, at least in part, from local glucocorticoid metabolism. Although this mechanism has not yet been demonstrated in wolves, these findings provide additional support for the simultaneous determination of CORT and CORN and point to an important area for future research on glucocorticoid metabolism in hair follicles and skin of wild species.

## 3. Materials and Methods

### 3.1. Materials and Reagents

Chemicals of the highest available purity were used. LC-MS-grade water and methanol were obtained from VWR (Radnor, PA, USA). Isopropanol, acetic acid, and formic acid, all of LC-MS grade, were purchased from Merck (Darmstadt, Germany). Hormone standards, including CORT and isotopically labelled CORT-D_4_, as well as CORN and isotopically labelled CORN-D_8_, were obtained from Merck (Darmstadt, Germany). Methanolic working solutions were stored at −20 °C. Hair extracts were purified using Strata-X SPE cartridges (30 mg sorbent, p/n 8B-S100-TAK) purchased from Phenomenex (Aschaffenburg, Germany).

### 3.2. Origin of Hair Samples

The samples were collected in Poland between 2014 and 2024 from wolves that had died, e.g., in vehicle collisions. Hair was sampled from the dorsal body region and did not contain follicles. For each individual, sex was determined based on external morphological traits, age was estimated from tooth wear [60], and geographic origin was recorded (Table 4). The sampled wolves originated from north-western, south-western, and south-eastern Poland. After collection and description, hair samples were stored in paper envelopes in the dark at room temperature until analysis.

### 3.3. Hair Pretreatment and Homogenization

**Figure 3:**
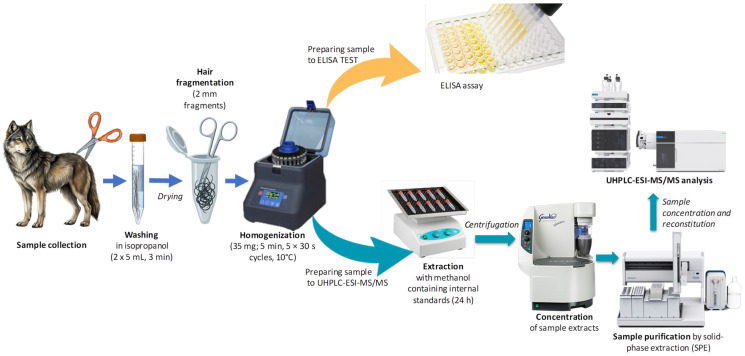
Flowchart illustrating the hair sample pretreatment and analytical procedure.

Hair samples were manually shaken with 5 mL of isopropanol for 3 min to remove surface contamination [22]. This step was repeated twice using fresh isopropanol each time. The hair samples were then left to dry overnight at room temperature. After drying, the hair was cut into approximately 2 mm fragments using surgical scissors. Next, 35 mg of each sample was weighed using an XA 100.3Y.A balance (Radwag, Radom, Poland) into plastic Falcon tubes designated for homogenization. The samples were then ground with five steel balls in a Bioprep-24R Homogenizer (EnviScience, Warsaw, Poland) for 5 min, consisting of five 30 s grinding cycles (and intervals between cycles) at 10 °C with cooling. The powdered hair samples were subsequently used for UHPLC-ESI-MS/MS analysis and ELISA. The applied workflow is presented in Figure 3.

### 3.4. Preparation of Hair Samples for CORT and CORN Determination by UHPLC-ESI-MS/MS

Samples were prepared according to a previously optimized protocol with minor modifications [28]. The powdered hair samples were incubated in 1 mL of methanol containing isotopically labelled internal standards (15 ng/mL of CORT-D_4_ and CORN-D_8_) for 24 h using a SSL4 see-saw rocker (Stuart, Vernon Hills, IL, USA) at 70 rpm and room temperature. Next, the samples were centrifuged for 10 min at 13,000 rpm using a 5415R centrifuge (Eppendorf, Hamburg, Germany). After centrifugation, 800 µL of the supernatant was collected, transferred to a new tube, and evaporated to dryness in an EZ-2 Elite Personal Evaporator (Genevac Limited, Ipswich, UK). Subsequently, the dried residue was reconstituted in 1 mL of LC-MS-grade-water and ultrasonicated for 2 min in an ultrasonic bath (Polsonic, Warsaw, Poland). The prepared samples were purified by SPE using a Gilson GX-271 ASPEC automated extraction system (Middleton, WI, USA). The system was controlled using Gilson Ethernet Utility 1.8.6.1 and Trilution LH software 2.0. Before sample loading, Strata-X SPE cartridges were conditioned with 1 mL of methanol and equilibrated with 1 mL of water. Then, 1 mL of the sample was loaded onto the cartridges. The cartridges were washed with 1 mL of water, and the analytes were eluted with 1 mL of methanol. The eluate was concentrated to 200 µL, transferred to a glass chromatography vial, and analyzed by UHPLC-ESI-MS/MS in three replicate injections.

### 3.5. UHPLC-ESI-MS/MS Analysis of Hair Extracts

UHPLC-ESI-MS/MS analyses were carried out under conditions based on a previously developed method [27], with modifications. Separation of sample components was performed using a 1290 Infinity UHPLC system consisting of a degasser, binary pump, autosampler, and column thermostat (Agilent Technologies, Santa Clara, CA, USA). The UHPLC system was coupled to an Agilent 6460 triple quadrupole (QQQ) mass spectrometer equipped with an Agilent Jet Stream ESI source. Data acquisition was performed using Agilent MassHunter Acquisition vB10.1, while data analysis was carried out using Agilent MassHunter Quantitative Analysis vB12.1 software.

Chromatographic separation was performed at 40°C on a BEH C18 1.7 µm VanGuard Fit column (2.1 × 100 mm, Waters, Milford, CT, USA). The mobile phase consisted of 0.1% (*v*/*v*) formic acid in water (solvent A) and methanol (solvent B) with the flow rate set at 0.2 mL/min. The gradient elution programme was as follows: 0–4 min, 20–60% solvent B; 4–5 min, 60% solvent B; 5–7 min, 60–70% solvent B; 7–9 min, 70% solvent B (post run: 2 min). The injection volume was 5 µL.

Data were acquired in dMRM mode. Ions were monitored in positive ionization mode (settings detailed in Table 5). MS source conditions were based on a previous study [28] and were as follows: nebulizer pressure, 35 psi; drying gas temperature, 300 °C; drying gas flow, 8 L/min; sheath gas temperature, 300 °C; sheath gas flow, 10 L/min; capillary voltage, 4500 V, and nozzle voltage, 300 V.

### 3.6. Determination of CORT by ELISA-Sample Preparation and Analysis

To compare the LC-MS/MS results with those obtained using an independent analytical approach, CORT concentrations were measured in nine wolf hair samples using a commercial ELISA kit (Cortisol ELISA Kit, Cayman Chemical, Ann Arbor, MI, USA; Cat. No. 500360), intended for cortisol determination in various biological matrices, including hair. The assay was performed according to the manufacturer’s instructions. Briefly, 35 mg of powdered hair from each sample was weighed into a tube, mixed with 1.8 mL of methanol, vortexed, and incubated overnight on an orbital shaker. The tubes were then centrifuged, and the recovered supernatant was evaporated to dryness under a gentle stream of nitrogen at 37 °C. The dried extracts were reconstituted in 400 µL of ELISA Buffer, vortexed, and used for CORT quantification according to the manufacturer’s protocol. The wells were emptied and washed using an ELx50 microplate strip washer (BioTek Instruments, Inc., USA), incubated using an ELMI DTS-4 digital thermostatic microplate shaker (ELMI SIA, Riga, Latvia), and read at 410 nm using a Synergy 2 multi-mode microplate reader (BioTek Instruments, Inc., Winooski, VT, USA). Raw absorbance data were blank-corrected, and the ratio of sample absorbance to maximum binding, expressed as B/B_0_, was calculated. Cortisol concentrations obtained from the calibration curve were initially expressed as pg/mL and then converted to pg/mg of hair, taking into account the reconstitution volume and the initial hair mass.

### 3.7. UHPLC-ESI-MS/MS Method Validation

For validation purposes, a representative hair matrix was prepared by pooling wolf hair samples obtained from multiple individuals and characterized by low endogenous levels of the target hormones (preselected by an ELISA test). This pooled matrix was used to prepare a series of QC samples for the comprehensive evaluation of method performance. The following validation parameters were assessed: LOD, LLOQ, linearity, accuracy, precision, extraction recovery (R_E_), matrix effect (ME), and analyte stability. Stability was evaluated using two different tests: autosampler stability (S_A_) and freeze–thaw stability (S_FT_). The validation procedure was performed in accordance with FDA guidelines [61].

#### 3.7.1. Method LOD, LLOQ, and Linearity

Linearity was evaluated using matrix-matched calibration curves prepared in the pooled grey wolf hair matrix. The calibration set consisted of nine samples containing a fixed concentration of internal standards: CORT-D_4_ and CORN-D_8_ (15 ng/mL each). The calibration range extended from 0.144 ng/mL for CORT and 0.087 ng/mL for CORN to 0.1 µg/mL for both analytes. Detailed concentration levels of the analytes in the matrix-matched calibration solutions are provided in Supplementary Table S1. All calibration samples were analyzed in triplicate, and mean values were used to construct calibration curves. In addition, blank matrix samples containing only ILIS (without added analytes) were analyzed to estimate the endogenous levels of CORT and CORN in the grey wolf hair matrix. When endogenous signals were detected, they were subtracted from the responses obtained for the calibration standards. Calibration solutions were prepared according to the developed sample preparation protocol used for study samples to ensure methodological consistency. Based on the resulting matrix-matched calibration curves, LOD and LLOQ values were calculated as the standard deviation of the intercepts (*n* = 3) divided by the slope of the calibration function and multiplied by 3.3 or 10, respectively. Coefficient of determination (R^2^)of 0.99 or greater were required to confirm linearity.

#### 3.7.2. Method Precision and Accuracy

Accuracy and precision were evaluated using QC samples fortified with the analytes at three concentration levels: low (LQC, at approximately the LLOQ), medium (MQC, corresponding to 50% of the upper calibration limit, 50 ng/mL), and high (HQC, corresponding to 80% of the upper calibration limit, 80 ng/mL). Intraday accuracy and precision were assessed using five independently prepared QC samples analyzed within a single day, whereas interday accuracy and precision were evaluated using 15 independently prepared QC samples analyzed across three different days. This approach enabled a comprehensive evaluation of method performance across the analytical range. Precision was expressed as the relative standard deviation (RSD, %) and calculated as (*SD*/*M*) × 100%, where *SD* represents the standard deviation, and *M* is the mean analyte concentration calculated from the calibration curve. Accuracy was determined as the ratio of the measured concentration (*M*) to the nominal (true) concentration (*T*), expressed as a percentage: accuracy = *M*/*T* × 100%. Results were considered acceptable when precision was ≤15% for MQC and HQC and ≤20% for LQC, and when accuracy was within 85–115% for MQC and HQC and within 80–120% for LQC.

#### 3.7.3. Recovery and Matrix Effect

R_E_ and ME were evaluated at three concentration levels: LQC, MQC, and HQC. RE was calculated as the ratio of the analytical responses obtained for matrix samples spiked with the analytes before extraction (*B*) to those obtained for matrix extracts spiked with the analytes at the final stage of the sample preparation procedure (*C*), expressed as a percentage according to the equation: R_E_ = *B*/*C* × 100%. R_E_ was considered acceptable when it ranged from 70 to 120%. ME was evaluated by comparing the analyte responses obtained for solvent-based standards without matrix (*D*) with those obtained for matrix extracts spiked with the analytes at the final stage of sample preparation, after subtraction of endogenous glucocorticoid signals determined in blank matrix samples (*E*). ME was calculated based on analytical responses using the formula ME = (*E*/*D*) × 100%.

#### 3.7.4. Stability Evaluation

Stability was assessed by comparing the analyte responses obtained from freshly prepared hair samples with those obtained after 24 h storage at 25 °C in an autosampler tray (S_A_). Freeze–thaw stability (S_FT_) was evaluated after three freeze–thaw cycles between −20 and +25 °C. Stability was considered acceptable when the deviation from freshly prepared samples did not exceed ±10% for MQC and HQC and ±15% for LQC.

## 4. Conclusions

The present study provides the first validated UHPLC-ESI-MS/MS method for the simultaneous determination of CORT and CORN in grey wolf hair. The method showed satisfactory validation parameters and was successfully applied to hair samples from wild-living wolves, confirming its suitability for glucocorticoid analysis in this wildlife-relevant matrix. CORT concentrations determined by UHPLC-ESI-MS/MS showed a strong positive correlation with ELISA results, supporting the applicability of the developed protocol while also emphasizing the greater selectivity of the mass spectrometry-based approach. Importantly, unlike ELISA, the proposed method enables the simultaneous determination of CORN, thereby providing broader insight into glucocorticoid metabolism and stress-related physiological processes. Overall, this method represents a useful analytical tool for future studies on stress physiology and welfare assessment in wild carnivores.

## Figures and Tables

**Table 1:** Calibration and validation parameters for cortisol (CORT) and cortisone (CORN).

Parameter	CORT	CORN
Regression equation	*y* = 0.136*x* + 0.010	*y* = 0.093*x* + 0.007
Variables	*y*—peak area ratio (CORT/CORT-D_4_); *x*—analyte concentration (ng/mL)	*y*—peak area ratio (CORN/CORN-D_8_); *x*—analyte concentration (ng/mL)
R^2^	0.994	0.999
Linear range	0.144 ng/mL–0.1 µg/mL (4.13 pg/mg–2.86 ng/mg)	0.087 ng/mL–0.1 µg/mL (2.77 pg/mg–2.86 ng/mg)
LOD	47.66 pg/mL (1.36 pg/mg)	28.78 pg/mL (0.82 pg/mg)
LLOQ	0.144 ng/mL (4.13 pg/mg)	78.2 pg/mL (2.49 pg/mg)

LLOQ—lower limit of quantification; LOD—limit of detection; R^2^—coefficient of determination.

**Table 2:** Validation parameters of the developed method.

	CORT	CORN
Intraday precision [RSD,%] (*n* = 5)		
LQC	5.7	9.6
MQC	8.2	3.2
HQC	3.5	5.1
Interday precision [RSD,%] (*n* = 15)		
LQC	13.0	8.1
MQC	6.6	11.4
HQC	6.6	8.8
Intraday accuracy [%] (*n* = 5)		
LQC	101.0	106.3
MQC	101.6	107.8
HQC	108.0	106.0
Interday accuracy [%] (*n* = 15)		
LQC	107.7	109.3
MQC	108.4	100.9
HQC	105.1	102.0
Extraction recovery (R_E_) [%] (*n* =2)		
LQC	77.0 ± 0.7	84.5 ± 2.2
MQC	77.4 ± 1.9	80.0 ± 0.2
HQC	81.9 ± 0.9	96.4 ± 3.1
Matrix effect (ME) [%] (*n* =2)		
LQC	97.4 ± 3.7	92.6 ± 3.2
MQC	97.4 ± 3.7	100.9 ± 2.7
HQC	92.3 ± 2.0	93.8 ± 1.5
Autosampler stability (S_A_) [%]		
LQC	93.7	93.9
MQC	94.4	96.4
HQC	96.0	98.4
Freeze–thaw stability (S_FT_) [%] (*n* =2)		
LQC	97.1 ± 0.3	104.8 ± 3.5
MQC	91.1 ± 0.7	90.6 ± 2.3
HQC	95.9 ± 2.0	103.3 ± 6.4

LQC, MQC, and HQC refer to low-, medium-, and high-concentration quality control samples, respectively.

**Table 3:** Cortisol (CORT) and cortisone (CORN) levels determined in wolf hair samples using UHPLC-ESI-MS/MS and ELISA CORTISOL assay.

Sample Number	CORT ± SD [pg/mg]	CORN ± SD [pg/mg]	ELISA CORT [pg/mg]
H_21	<LLOQ (<4.13)	<LLOQ (<2.49)	3.10 ± 0.14
H_101	<LLOQ (<4.13)	<LLOQ (<2.49)	3.85 ± 0.04
H_185	<LLOQ (<4.13)	<LLOQ (<2.49)	3.73 ± 0.09
H_39	4.26 ± 0.26	<LLOQ (<2.49)	8.21 ± 2.28
H_122	4.28 ± 0.04	3.67 ± 0.09	10.30 ± 0.53
H_260	4.22 ± 0.03	<LLOQ (<2.49)	9.24 ± 0.48
H_18	11.86 ± 0.89	2.52 ± 0.24	35.32 ± 3.45
H_97	8.69 ± 0.25	3.51 ± 0.33	40.83 ± 1.41
H_200	10.90 ± 0.57	2.62 ± 0.36	33.60 ± 0.57

<LLOQ—analyte was detected but not quantified. Standard deviation (SD) was calculated from three injections of the same sample.

**Table 4:** Characteristics of grey wolf hair samples included in the study.

Sample ID	Sex	Age Class	Place of Origin (Forest District Name)	Sampling Date
H_21	Male	Adult	Gościno	5 January 2014
H_101	Female	Adult	Karwin	28 July 2018
H_185	Female	Juvenile	Czarne Człuchowskie	27 November 2019
H_39	Female	Juvenile	Żagań	31 December 2021
H_122	Female	Juvenile	Świebodzin	8 April 2017
H_260	Female	Adult	Międzychód	22 December 2023
H_18	Male	Adult	Lubin	8 May 2021
H_97	Female	Juvenile	Bobolice	8 November 2023
H_200	Female	Juvenile	Lutowiska	28 November 2022

**Table 5:** Instrumental settings for the dMRM method used for the quantitative determination of CORT and CORN in positive ionization mode.

Analyte	Precursor ion (*m*/*z*)	Product Ion (*m*/*z*)	Fragmentor Voltage [V]	Collision Energy [eV]	Retention Time [min]	ΔRetention Time [min]
CORN	361.1	163/121	160	24/36	6.3	4
CORN-D_8_	369.1	169.1	160	30	6.3	4
CORT	363	121.2/97.5	80	26/36	6.8	4
CORT-D_4_	367.1	121.1	80	26	6.8	4

Abbreviations: CORN—cortisone; CORN-D_8_—deuterated cortisone internal standard; CORT—cortisol; CORT-D_4_—deuterated cortisol internal standard.

## Data Availability

The data supporting the findings of this study are available within the article and its Supplementary Materials. Additional data are available from the corresponding author upon reasonable request.

## Supplementary Materials

The following supporting information can be downloaded at: https://www.mdpi.com/article/10.3390/molecules31142420/s1, Table S1. Concentrations of cortisol (CORT) and cortisone (CORN) in calibration standards. Figure S1. Representative matrix-matched calibration curves for cortisol (CORT) and cortisone (CORN).
