# Supplementary material for: A Validated LC-MS/MS Method for Simultaneous Determination of Cortisol and Cortisone in Grey Wolf Hair for Application in Ecological Studies
Source: Molecules. 2026 Jul 10;31(14):2420. doi: 10.3390/molecules31142420 (PMC13413905; doi:10.3390/molecules31142420)

A Validated LC-MS/MS Method for Simultaneous Determination of Cortisol and Cortisone in Grey Wolf Hair for Application in Ecological Studies

Arkadiusz Jastrzębski [1], Kinga Ożga-Wybranowska [1], Rafał Łopucki [1], Sabina Nowak [2,3], Robert W. Mysłajek [2,3] and Ilona Sadok [4,*]

[1] Department of Biomedicine and Environmental Research, Institute of Biological Sciences, Faculty of Medicine, The John Paul II Catholic University of Lublin, Konstantynów 1J, 20-708 Lublin, Poland; arkadiusz.jastrzebski@kul.pl (A.J.); kinga.ozga@kul.pl (K.O.-W.); lopucki@kul.pl (R.Ł.)

[2] Department of Animal Ecology and Evolution, Institute of Ecology, Faculty of Biology, University of Warsaw, Żwirki i Wigury 101, 02-089 Warszawa, Poland; s.pieruzek-nowak@uw.edu.pl (S.N.); r.myslajek@uw.edu.pl (R.W.M.)

[3] Association for Nature "Wolf", Cynkowa 4, 34-324 Twardorzeczka, Poland

[4] Department of Biomedical and Analytical Chemistry, Institute of Biological Sciences, Faculty of Medicine, The John Paul II Catholic University of Lublin, Konstantynów 1J, 20-708 Lublin, Poland

[*] Correspondence: ilona.sadok@kul.pl; Tel.: +48-81-445-46-18

Arkadiusz Jastrzębski: arkadiusz.jastrzebski@kul.pl; ORCID: 0009-0006-9079-6541

Kinga Ożga-Wybranowska: kinga.ozga@kul.pl; ORCID: 0000-0001-5152-3555

Rafał Łopucki: lopucki@kul.pl, ORCID: 0000-0003-2137-8742

Sabina Nowak: s.pieruzek-nowak@uw.edu.pl; ORCID 0000-0002-7771-8032

Robert W. Mysłajek: r.myslajek@uw.edu.pl; ORCID 0000-0001-9619-2868

Ilona Sadok: ilona.sadok@kul.pl; ORCID: 0000-0003-1154-7581

Supplementary Table S1. Concentrations of cortisol (CORT) and cortisone (CORN) in calibration standards.

CORN	0.087; 0.3; 0.5; 1.5; 5; 10; 50; 80; 100 ng/mL
CORT	0.144; 0.3; 0.5; 1.5; 5; 10; 50; 80; 100 ng/mL

Supplementary Figure S1. Representative matrix-matched calibration curves for cortisol (CORT) and cortisone (CORN).

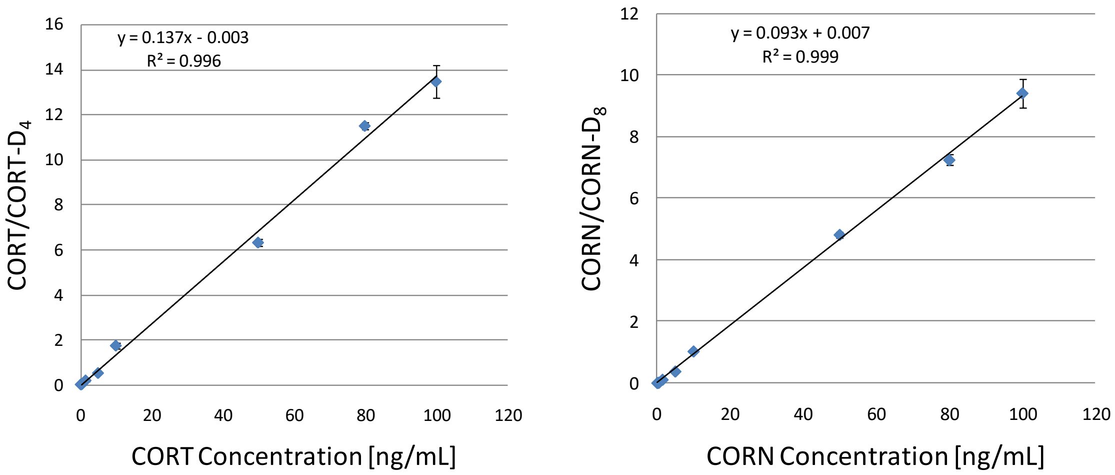

Supplement: Supplementary file 1 [file molecules-31-02420-s001.zip › molecules-4406300-supplementary.pdf]
